# First-Principles Calculations of Structural, Electronic, Optical, Vibrational, Dielectric, and Elastic Properties of Tetragonal Mg_2_TiO_4_ and Mg_2_TiO_4_:Mn^4+^

**DOI:** 10.3390/nano16191213

**Published:** 2026-09-25

**Authors:** Leonid L. Rusevich, Eugene A. Kotomin, Miroslav D. Dramićanin, Alok M. Srivastava, Mikhail G. Brik

**Affiliations:** 1 Institute of Solid State Physics, University of Latvia, Kengaraga Street 8, LV-1063 Riga, Latvia; kotomin@latnet.lv; 2 Centre of Excellence for Photoconversion, Vinča Institute of Nuclear Sciences—National Institute of the Republic of Serbia, University of Belgrade, 11000 Belgrade, Serbia; dramican@vinca.rs; 3 Current Chemicals Inc., 1099 Ivanhoe Road, Cleveland, OH 44110, USA; srivastaam@outlook.com; 4 Institute of Physics, University of Tartu, W. Ostwald Str. 1, 50411 Tartu, Estonia; 5 School of Integrated Circuits, Chongqing University of Posts and Telecommunications, Chongqing 400065, China; 6 Academy of Romanian Scientists, 3 Ilfov, 050044 Bucharest, Romania

**Keywords:** LED, phosphors, Mg_2_TiO_4_, inverse spinel, dopant, first-principles calculations, DFT, atomic and electronic structure, vibrational, dielectric and elastic properties, Raman and IR spectra

## Abstract

In this study, first-principles simulations of inverse spinel Mg_2_TiO_4_ (MTO) and MTO:Mn^4+^ on the base of the tetragonal phase were performed within the linear combination of atomic orbitals (LCAO) approximation of density functional theory (DFT), as implemented in CRYSTAL23 computer code. The effect of Mn doping on the MTO band gap structure was studied for the first time. Lattice relaxation around Mn^4+^ ions upon substitution was considered. Two possible spin configurations of the Mn^4+^ ion (3d^3^) in MTO:Mn^4+^ (total spin of system *S* = 3/2 and 1/2) were considered in the framework of the unrestricted (open shell) DFT-LCAO calculations. The structural, electronic, vibrational, dielectric and elastic properties of materials were investigated, compared and discussed. It has been shown that dopant atoms create new defect states within the band gap and modify the conduction band and the valence band, which is one of the most important results in the present study. The effect of isotopic substitution on the vibrational properties was analyzed. Based on the calculated elastic moduli, the Debye temperature for MTO was estimated. This study provides a basis for the assessment of the optical and luminescent properties of the doped material and its use in LED applications.

## 1. Introduction

Crystalline solids doped with tetravalent manganese ions are widely used for lighting applications as red phosphors that convert blue or ultraviolet excitation into red emission arising from the Mn^4+^ ^2^E_g_ → ^4^A_2g_ spin-forbidden transition [1,2,3,4,5]. Addition of such red phosphors to white LEDs drastically improves their characteristics, such as correlated color temperature and color rendering index [6,7].

It has been demonstrated that the remarkably wide variation in the Mn^4+^ emission wavelength in various hosts (from 620 nm in K_2_SiF_6_ to 723 nm in SrTiO_3_) is due to the covalent effects between Mn^4+^ ions and ligands, which are essentially host-dependent [8,9]. Therefore, detailed theoretical investigations of structural, electronic, optical, elastic and thermodynamic properties of these materials, pure and Mn^4+^-doped, are an important task, which can be tackled by modern density functional theory (DFT) computational methods.

Consistent calculations of the above-mentioned properties and their meaningful comparison across different compounds helps to understand materials’ behavior and uncover various trends that can be useful in the search for new phosphor candidate materials with improved performance.

Recently, we calculated and compared properties of some hexafluorosilicate compounds used in commercial LEDs [10,11,12]. In the present paper, we consider another material, magnesium orthotitanate, Mg_2_TiO_4_ (MTO), with different anions—oxygen, not fluorine. The covalent effects in the oxide materials are more pronounced compared to highly ionic fluorides. MTO is an example of an inverse spinel whose physical properties depend on the distribution of cations in the crystal lattice. There are two types of cation sites in the spinel structure—tetrahedrally and octahedrally coordinated—and the number of octahedral sites is twice the number of tetrahedral ones. In fully inverse spinel, the octahedral sites are occupied by both types of cations (Mg^2+^ and Ti^4+^ in the case of MTO) in a 1:1 ratio, and the tetrahedral sites are solely occupied by one type of cation (Mg^2+^ in MTO). At the same time, in inverse spinel, two different types of cations occupying octahedral sites can be either ordered or disordered. The structure of the material can vary depending on the synthesis conditions, but, in general, MTO crystallizes in two different modifications: high temperature (cubic space group sg227, *Fd-3m*) and low temperature (tetragonal sg91, *P4_1_22*). The structural transition between phases occurs in the temperature range of 660–950 °C [13,14]. The tetragonal phase is considered as a stable and ordered form. In this phase, the Ti^4+^ ions and half of the Mg^2+^ ions are located in octahedral sites at different Wyckoff positions (4b and 4a, respectively), the second half of the Mg^2+^ cations occupy 4c positions in tetrahedral sites, and two sets of oxygen anions are located in 8d_1_ and 8d_2_ Wyckoff positions. On the other hand, the cubic phase is disordered. It is characterized by a statistical distribution of Mg^2+^ and Ti^4+^ cations in octahedral sites. These ions are randomly located in a single Wyckoff position (16d), the Mg^2+^ cations occupy the 8a position in tetrahedral sites, and the oxygen ions are in the 32e Wyckoff position. The crystallographic unit cell of MTO in the tetragonal phase contains four formula units (28 atoms), whereas in the cubic phase it contains eight formula units (56 atoms). Although both structural modifications have been the subject of research, the main focus so far has been on the cubic phase.

Analysis of the available literature on MTO reveals several earlier publications in which some aspects of this material are described. Thus, the phase transition from the high-temperature cubic to the low-temperature tetragonal structure was studied in Ref. [14]. Quantum-mechanical atomistic calculations of the cation disorder phenomena in the spinel structure of MTO were reported in Ref. [15], whereas the energies and disorder of different spinels including MTO were thoroughly calculated and analyzed in Ref. [16]. A discrete variation multielectron method was used in Ref. [17] to calculate energy levels of V^2+^, Cr^3+^, and Mn^4+^ in MTO. Zeng et al. [18] used the plane-wave-based CASTEP code to calculate the structural, elastic, mechanical, electronic, optical properties of cubic MTO modification, whereas Kushwaha et al. [19] analyzed the structural, elastic, vibrational, and thermodynamic properties of both cubic and tetragonal MTO phases. Distribution of the Mg and Ti cations in this compound was modeled in Ref. [20]. Experimental investigations of MTO potential as a host material for red phosphors were presented in Ref. [21]. DFT-based search for potential sensitizers to enhance emission of Mn^4+^ in MTO was described in Ref. [22], whereas experimental studies of influence of cation codopants on the intensity of the Mn^4+^ emission in this host were outlined in Ref. [23]. Experimental spectroscopic studies of cubic MTO:Mn^4+^ nanoparticles were reported in Ref. [24], along with the crystal field calculations of the Mn^4+^ energy levels.

In this paper, we continue our research on the properties of MTO by adding first-principles calculations of its structural, electronic, optical, and elastic properties in the tetragonal phase. In addition, we modeled the effects of manganese doping, replacing one Ti^4+^ ion by a Mn^4+^ ion, paying thorough attention to the local distortions around the substituted ion and the appearance of additional energetical states associated with impurity in the host’s band gap.

Section 2 contains all details of calculations, whereas the obtained results are presented in Section 3. The main conclusions of the calculations presented here are summarized in Section 4.

## 2. Computational Details

The computational methods used in this study are described in detail in our earlier paper [10]. Here, we provide a brief overview, focusing on the differences, key aspects and specific details relevant to the present calculations.

The first-principles (ab initio) simulations of pure MTO in the tetragonal phase (sg91, *P4_1_22*) and MTO:Mn^4+^ were performed within the linear combination of atomic orbitals (LCAO) approximation of density functional theory (DFT), as implemented in CRYSTAL23 computer code [25,26]. In the computations, the tetragonal phase of MTO was considered as a fully inverse spinel with complete cationic ordering, where Ti^4+^ and Mg^2+^ ions in octahedral sites are located in different Wyckoff positions (4b and 4a, respectively), Mg^2+^ cations in tetrahedral sites occupy 4c positions, and two sets of oxygen anions are located in 8d_1_ and 8d_2_ Wyckoff positions. The crystallographic unit cell of MTO in this phase is identical to the primitive unit cell and contains 8 Mg, 4 Ti and 16 oxygen atoms (four formula units).

The spin of the defective system MTO:Mn^4+^ (the substitution of one of the four Ti atoms by a Mn atom) is determined by the spin configuration of the Mn^4+^ ion. The Mn^4+^ ion has three unpaired electrons (3d^3^). In simulation of the systems with unpaired spins, all electrons are divided into two groups: those with spin up (α-electrons) and those with spin down (β-electrons). The total spin of an atom (and the system as a whole) depends on the difference in the number of α- and β-electrons. Therefore, two possible total spin states (*S* = 3/2 and 1/2) of MTO:Mn^4+^ were considered within the framework of spin-polarized unrestricted (open shell) DFT-LCAO calculations [25].

The appearance of a dopant in the system leads to a decrease in symmetry and splitting of the Wyckoff positions. In our case, the symmetry of the defective system corresponds to the monoclinic sg3 (*P2*), and the 4b position of Ti atoms in pure crystal splits into 1c, 1d and 2e Wyckoff positions in crystal with dopant. In the calculations, the Mn atom was located in the 1c position.

We performed preliminary calculations of the structural properties and band gap of a perfect MTO to determine the optimal combination of basis sets and functionals for the simulation of this material. Three different kits of Gaussian basis sets (including the TZVP_2012 kit, which was employed in previous studies of K_2_SiF_6_ and Cs_2_SiF_6_ [10,11]) and three hybrid exchange-correlation functionals (PBE0, B1WC, and WC1LYP [25]) were used in the test calculations. After comparing the obtained results with the available experimental data, we concluded that the best combination of basis sets and functionals is the TZVP_rev2 basis sets and B1WC functional. Thus, the Mg, Ti, O and Mn atoms are described in our calculations by all-electron TZVP_rev2 basis sets [27] (these basis sets are also available on the CRYSTAL23 Basis Sets Library website [28]). The single-parameter hybrid B1WC functional combines the Wu–Cohen WCGGA exchange functional with 16% (by default) of the exact Hartree–Fock (HF) exchange and the Perdew–Wang PWGGA correlation functional [25,29]. Its application to ideal and doped crystals has been tested before in our earlier publications [10,11] to yield good agreement of the calculated structural and electronic properties with the experimental ones.

In the present study, simulations of structural, electronic, vibrational, dielectric and elastic properties of MTO and MTO:Mn^4+^ were performed. The analysis of all properties was fulfilled on the basis of the fully optimized geometry of systems. The frequencies of the transverse optical (TO) vibrational modes and vibrational contribution to complex dielectric function *ε*(*ν*) = *ε_el_* + *ε_vib_*(*ν*) were calculated for the center of the first Brillouin zone (the Γ-point) within the harmonic approximation. The complex dielectric function is the sum of the electronic (high-frequency) *ε_el_* and ionic (vibrational) *ε_vib_*(*ν*) components calculated in CRYSTAL code based on a classical dispersion relation of Drude–Lorentz model [25,30]. The electronic part is calculated through the Coupled Perturbed Hartree–Fock/Kohn–Sham (CPHF/CPKS) scheme, adapted for periodic systems [31,32]. To analyze vibrational modes, the isotopic substitution method (change in the relative atomic masses of individual atoms) was applied [10,11,25,33,34]. The IR and Raman integrated intensities for vibrational modes were calculated; the one-phonon IR absorbance and Raman spectra were also simulated. For verification, the intensity of IR-active modes was calculated using two methods: the Berry phase approach (with numerical differentiation) and the CPHF approach, which is fully analytical [25,35]. The relative intensities of Raman-active modes (normalized to the maximum intensity of Raman-active modes) were calculated using a fully analytical method [36,37]. Electronic *ε_el_* and vibrational *ε_vib_*(0) contributions to the static dielectric tensor *ε*(0) were estimated.

Symmetric elastic tensors, as well as bulk modulus (*B*), shear modulus (*G_H_*), Young’s modulus (*E_H_*), and Poisson’s ratio (*ν_H_*) in the Hill averaging scheme were calculated for perfect and defective materials. It should be noted that for better accuracy when calculating the elastic parameters, we used a five-point scheme for numerical calculations of the second derivative (NUMDERIV = 5; default setting is 3). We also add that during computations of all properties for perfect crystal, the shrinking factor (SHRINK) was set to 8, and for crystal with the dopant (calculations with spin), SHRINK was equal to 6 [25]. Other technical details of the calculations can be found in Ref. [10].

## 3. Results and Discussion

### 3.1. Structural Properties

The atoms in the tetragonal unit cell of MTO are located in the following Wyckoff positions: Mg__tetr_: 4c(*x*,*x*,3/8) (Mg in tetrahedral sites); Mg__oct_: 4a(0,*y*,0) (Mg in octahedral sites); Ti: 4b(1/2,*y*,0) (octahedral sites); oxygen atoms: 8d(*x*,*y*,*z*). In this case, there are two groups of oxygen atoms (O1 and O2) in the unit cell, located in positions 8d_1_ and 8d_2_ (total number of oxygen atoms in unit cell is 16). The initial lattice constants and atom positions for the calculation were taken from the experimental data in Ref. [14]. Table 1 presents the calculated variable parts of atomic coordinates after full geometry optimization in comparison with experimental values [14] for perfect tetragonal MTO.

Figure 1 demonstrates the simulated unit cell of the perfect 28-atom tetragonal MTO which is used in the calculations. All structural figures in the paper are drawn with VESTA software (ver. 3.4.4) [38].

Figure 2 shows in more detail a part of the crystal structure of MTO—oxygen octahedra with Mg and Ti atoms in the center and an oxygen tetrahedron with a Mg atom in the center. Interestingly, all cations have the same local symmetry—monoclinic *C_2_*(*2*). Additionally, Figure 2 reveals the difference between the O1 and O2 atoms in the tetragonal MTO structure. Both O1 and O2 atoms are bonded to four cations: one Mg cation in tetrahedral coordination and three more cations in octahedral coordination. However, the O1 atom is connected with two Mg cations and one Ti cation, whereas the O2 atom is bonded to one Mg and two Ti cations in octahedral positions. Therefore, the O1 and O2 atoms are not equivalent in symmetry. The local symmetry of the positions of the O1 and O2 atoms corresponds to the point group *C_1_*(*1*), i.e., there is no symmetry at all, and the environment of each oxygen atom is completely asymmetric.

Calculated values of the structural parameters of the perfect MTO along with corresponding experimental data [13,14], as well as the discrepancies between calculated and measured values (in parentheses, as a percentage), are presented in Table 2.

In general, we see excellent agreement between the calculated and experimental values collected in Table 2. The discrepancies are less than 1% (in absolute value) for almost all parameters. Most of the calculated values in Table 2 are smaller than the corresponding experimental values. This can be explained by the fact that the calculations correspond to a temperature of 0 K and do not take into account thermal expansion. This fact further confirms the accuracy of our calculations. Let us now consider the results presented in Table 2 in detail.

In Table 2, *a*, *b* and *c* are the lattice constants of perfect tetragonal MTO. We also note the calculated lattice constants are close to those from Ref. [19] (*a* = *b* = 6.0281 Å, *c* = 8.4812 Å). In addition, close calculated lattice constants for the 28-atom unit cell of tetragonal MTO are given in Ref. [20] (*a* = *b* = 5.9758 Å, *c* = 8.3948 Å; calculations were performed by using the VASP computer code). Parameter *d* denotes the distance between cations and anions (bond length) in octahedra and tetrahedra, which is stated explicitly in the case of Mg as the central cation. The tetrahedron around the Mg cation is formed from two O1 and two O2 atoms (see Figure 2). In calculations, the distances *d*[Mg–O1]_tetr_ and *d*[Mg–O2]_tetr_ are very close to each other, but in experimental measurements they differ slightly more. Six oxygen atoms form octahedra around Mg and Ti cations. Namely, the oxygen octahedron around the Mg cation is formed from four O1 atoms and two O2 atoms, whereas the octahedron around the Ti is formed from two O1 atoms and four O2 atoms (see Figure 2). At the same time, among the four equivalent oxygen atoms in the octahedra (O1 in the Mg-octahedron and O2 in the Ti-octahedron), two pairs of atoms have slightly different distances from the central cation. To highlight this feature, we used letters “a” and “b” (e.g., O1a and O1b) in Figure 2 and Table 2. We emphasize here that these designations apply only to octahedra and are introduced solely to indicate the different distances of the corresponding oxygen atoms to the central cations of the octahedra. Moreover, in Figure 2, these designations apply exclusively to the highlighted octahedra, and in an adjacent octahedron with the same central cation, for example, the O2a atom would play the role of the O2b atom. Parameter <*d*> denotes the mean bond distance between the central cation and four or six oxygen atoms in the cases of tetrahedral or octahedral coordination, respectively. Examination of calculated bond lengths reveals that the shortest bond is *d*[Ti–O1] in the Ti-octahedron (this is consistent with the data from both experiments), and the largest bond is *d*[Mg–O2]_oct_ (see Table 2). The last statement is consistent with the data from Ref. [13] but contradicts the data from Ref. [14] (see Table 2). In Ref. [14], the value *d*[Mg–O1a]_oct_ is reported as the largest bond length, although the difference between *d*[Mg–O2]_oct_ and *d*[Mg–O1a]_oct_ in Ref. [14] is minimal. At the same time, the minimal mean bond length <*d*> is in the Ti-octahedron, but the maximal is in the Mg-octahedron, which completely coincides with the experimental data (see Table 2). Comparison of the octahedra volumes confirms this conclusion: *V_oct_*(Mg) > *V_oct_*(Ti). These results regarding mean bond length in octahedra and the volume of octahedra are quite expected. The Shannon ionic radius of the Mg^2+^ ion with coordination number 6 is 0.72 Å, whereas the Ti^4+^ ionic radius for coordination number 6 is 0.605 Å [39]. Note that mean cation–anion bond length <*d*[Mg–O]_tetr_> in tetrahedra also exceeds mean bond length <*d*[Ti–O]_oct_> in the Ti-octahedron (see Table 2), although the Shannon ionic radius of Mg^2+^ with coordination number 4 is 0.57 Å [39], i.e., less than the ionic radius of Ti^4+^ for coordination number 6.

So far, we have mainly focused on the position of the cations and their environment. Now let us briefly discuss the environment of the oxygen atoms O1 and O2. As noted earlier, both of these oxygen atoms are in tetrahedral coordination. The last four rows of Table 2 show the mean distances from the oxygen atoms to the four nearest cations and the volumes of the corresponding cationic tetrahedra around the oxygen atoms. Examination of these data reveals that the mean bond length and the volume of the surrounding tetrahedron are larger for the O2 atom, in full agreement with the experimental data.

Now consider a defective unit cell in which one Ti^4+^ cation is replaced by a Mn^4+^ cation (isovalent substitution; the dopant concentration is 25%). Although such a concentration of impurity is higher than in the experiments, we keep it for the analysis of local structures, local vibrational modes and local electronic properties around impurity. Realistic impurity concentration of about 1% would mean a super cell consisting of a 25-times increased unit cell, which would not be realistic from a computational point of view. Note that the Mn atom in Wyckoff position 1c of monoclinic sg3 keeps the same local symmetry (*C_2_*(*2*)) as the Ti atom in tetragonal sg91. A part of the crystal structure of MTO:Mn^4+^ is demonstrated in Figure 3, and Table 3 provides a comparison of selected structural parameters of the perfect system and two defective systems with different total spin states (*S* = 3/2 and *S* = 1/2).

Table 3 shows that the structural parameters of defective systems in different spin states differ little from each other. At the same time, the energy of the system (per unit cell) with *S* = 1/2 is 1.11 eV higher than the energy of the system with *S* = 3/2. This means that the ground state of MTO:Mn^4+^ has spin *S* = 3/2, in agreement with the energy level scheme derived from the crystal field calculations (the ground state is the spin-quartet orbital singlet ^4^A_2_). Therefore, in Section 3.2 we will continue to consider and compare the electronic properties of systems in different spin states, and in the subsequent subsection we will focus exclusively on the properties of the system with *S* = 3/2.

Regarding the changes in the MTO structure after the incorporation of the Mn dopant, Table 3 demonstrates that the lattice parameters and volume of the unit cell with the dopant are smaller than for the perfect unit cell (in particular, the volume decreases by slightly more than 1%). Table 3 also compares cation–oxygen distances, along with mean distances in the Ti-octahedron and Mn-octahedron in MTO and MTO:Mn, respectively. The results reveal that all parameters are smaller in the Mn-octahedron (the mean distance <*d*[Mn–O]_oct._> at *S* = 3/2 is less than <*d*[Ti–O]_oct._> by 2.7%). This conclusion also applies to volumes of Ti- and Mn-octahedra *V_oct._*(Ti{Mn}) in perfect and defective crystals, respectively (−7.4% for *S* = 3/2). The obtained results qualitatively correlate with the value of the Mn^4+^ ionic radius with coordination number 6, which is 0.53 Å [39], i.e., smaller than the Ti^4+^ ionic radius in octahedral coordination. The last two rows in Table 3 compare the oxygen–cation–(opposite (relative to the cation) oxygen) angles in Ti- and Mn-octahedra in perfect and defective systems, respectively. All octahedra in perfect MTO have a noticeable deviation from the ideal octahedral symmetry, so no such angle is equal to 180° (see Figure 2). The data in Table 3 reveal that these angles in the Mn-octahedron in MTO:Mn^4+^ are closer to 180° than in the Ti-octahedron in perfect MTO. Thus, the Mn substitution makes the Mn-octahedron less deformed compared to the Ti-octahedron in perfect MTO.

### 3.2. Electronic Properties

The band gap of perfect tetragonal MTO, obtained in our calculation, is 5.06 eV (direct transition in the Γ-point). The calculated electronic density of states (DOS) of such MTO is presented in Figure 4 (the figure exhibits total DOS and the contributions of Ti and O atoms, as well as separate contributions of Mg atoms in tetrahedra and octahedra). Figure 4 reveals that in the top of the valence band (VB), oxygen atoms make the dominant contribution to the electronic DOS, the contribution of Ti atoms is significantly smaller, and the contribution of Mg atoms is negligible. A specific feature of the conduction band (CB) of tetragonal MTO is the presence of sub-gaps where the density of states is equal to 0 (in the region of 6–7 eV and around 12 eV, Figure 4). The bottom of the CB consists mainly of Ti states with a small contribution of oxygen states. In the region of 10 eV (5 eV above the CB bottom), the states of O atoms and Mg atoms in the tetrahedra begin to play a major role, and only higher in the CB, after the second forbidden sub-gap, are Mg atoms in octahedra also “turned on”. Meanwhile, the contribution of Ti atoms is very small.

Note, that the authors of Ref. [20] report a value of 4.988 eV for the calculated band gap of tetragonal MTO. Moreover, they demonstrate the calculated DOS for this system (Figure 8 in Ref. [20]), and this DOS contains a forbidden sub-gap in the CB; however, the authors do not discuss this feature of DOS.

The calculated Mulliken effective charges [25] of ions in perfect MTO are as follows: *q*(Mg^2+^__tetr_) = 1.359|e| (charge of Mg ion in tetrahedron), *q*(Mg^2+^__oct_) = 1.211|e|, *q*(Ti^4+^) = 1.800|e|, *q*(O1^2-^) = −1.085|e|, and *q*(O2^2-^) = −1.100|e|. These values demonstrate that the interionic bonds in MTO are rather covalent in nature; the charges of all ions are quite close to 50% of their formal ionic charge. The Mg__tetr_ ion exhibits the most ionic nature, since its effective charge is 68% from its formal charge, whereas the effective charge of Mg__oct_ is only 61% from the formal charge, and the effective charge of Ti is just 45% from its formal charge. This indicates the highest degree of covalence in TiO_6_ complexes compared to other coordination polyhedra in MTO. Qualitative confirmation of this conclusion is provided by the values of electronegativity (Χ) of the elements: Χ(Mg) = 1.31, Χ(Ti) = 1.54, and Χ(O) = 3.44 according to the Pauling thermochemical scale [40]. After replacing the Ti^4+^ ion with the Mn^4+^ ion, the Mulliken charge of the oxygen ions and the remaining Ti^4+^ ions in MTO:Mn remain almost unchanged, while the charge of the Mn^4+^ ion is 1.606|e| (for *S* = 3/2) and 1.569|e| (for *S* = 1/2), indicating that the Mn–O bond becomes even less ionic than Ti–O bonds (Χ(Mn) = 1.55 [40]).

It should be stressed that the calculated values of the Mulliken charges depend on the choice of the basis set and, therefore, cannot be taken as an absolute reference. However, their values obtained within the same computational run with fixed computational settings for different ions in a crystal lattice can be considered as a reliable qualitative indicator that allows properties of chemical bonding between ions and peculiar features of electron density distribution in the space between them to be assessed.

To perform another independent analysis of chemical bonding in MTO, different from the Mulliken charges comparison, we have also applied the valence bond sum (VBS) theory outlined in Refs. [41,42]. Its application to various pairs of atoms in crystals can be found in Refs. [43,44].

The VBS of a given cation *i* surrounded by *n* anions enumerated by an index *j* can be obtained as follows:

(1) $$VBS=\;\sum_{j=1}^{n}exp\left(\frac{R_{0}-d_{ij}}{B}\right),$$

where $R_{0}$ is a constant for a given “cation–anion” pair (it can be found in Ref. [42]), $d_{ij}$ is the chemical bond length in the considered “cation–anion” pair, and *B* = 0.37 Å. The values of $R_{0}$ for the Mg^2+^–O^2-^, Ti^4+^–O^2-^ and Mn^4+^–O^2-^ pairs are 1.693, 1.815 and 1.753 Å, respectively. Using the optimized bond lengths from Table 2 and Table 3, the VBS values were calculated to be 1.829 for the tetrahedrally coordinated Mg^2+^, 2.118 for the octahedrally coordinated Mg^2+^, 4.095 for the octahedrally coordinated Ti^4+^, and 3.949 for the octahedrally coordinated Mn^4+^ (in the system with spin state *S* = 3/2). The VBS value of 1.829 for Mg^2+^ ions in the MgO_4_ tetrahedra is slightly lower than the formal valence +2 of magnesium ions, indicating the underbonded state (in other words, a decreased overlap of the manganese and oxygen wave functions and, as a consequence, a more ionic nature of the Mg–O bonds in this case); this conclusion agrees with the above-given analysis of the Mulliken charges. Moreover, values of VBS close to the formal charges of all cations confirm the stability of the studied compound and properly converged calculations of its optimized crystal structure.

During the full geometry optimization process, the resulting distribution of not only the charge but also the spin is determined. In our simulations, we observe full spin localization on the Mn^4+^ ion in optimized defective MTO:Mn systems. In the system with *S* = 3/2, the spin of the Mn ion is 2.98 electron spin, and in the system with *S* = 1/2, the spin of the Mn ion is 1.00, i.e., the calculations reveal that the entire spin of system in both cases is localized exclusively on the Mn ion.

The electronic DOSs of the defective MTO:Mn^4+^ systems with spins *S* = 3/2 and *S* = 1/2 are shown in Figure 5, Figure 6 and Figure 7. Figure 5a and Figure 6a present the total DOS and the contributions to the total DOS of the Mg, Ti, O and dopant Mn atoms for systems with *S* = 3/2 and *S* = 1/2, respectively. Figure 5b and Figure 6b present the total DOS and the projected DOS for the Mn atom only (to more clearly show the effect of the defect on DOS of the system) for systems with *S* = 3/2 and *S* = 1/2, respectively. Unlike Figure 4, in Figure 5a and Figure 6a, the projected DOS of Mg atoms demonstrates the total contribution of Mg atoms in tetrahedral and octahedral sites.

The dopant atoms create new defect states within the band gap and modify the VB and CB. Let us first consider the DOS of the MTO:Mn^4+^ system with *S* = 3/2 (Figure 5) and compare it with the DOS of perfect MTO (Figure 4). Overall, the band gap of the defective system with *S* = 3/2 is the same as in perfect MTO (~5.1 eV; CB in Figure 5a starts in the region where the Ti states give the main contribution to total DOS). The main feature of DOS at *S* = 3/2 is the presence of unoccupied manganese defect bands (both for α- and β-electrons) in the band gap, close to the bottom of the CB (0.39 eV and 0.24 eV below the CB for α- and β-electrons, respectively). These in-gap defect bands are formed primarily by the Mn states (see Figure 5), and their presence in the band gap is essential for the Mn^4+^ luminescence (details of which are beyond the scope of the present paper and will be considered in forthcoming publications). The minimum energy difference between the lowest defect levels and the top of the valence band is 4.1 eV for α-electrons (indirect transitions) and 4.6 eV for β-electrons (indirect transitions). In addition, the Mn atom produces new energy states in the CB in the 6.1–6.6 eV region, located in sub-gap where no energy states existed in perfect MTO. Note that dispersion of defect bands (their width) will be smaller at lower defect concentration, and therefore, the positions of the defect band may shift slightly. Finally, Figure 5 shows that the Mn atom gives some contribution to the upper part of the VB and the lower part of the CB. A more detailed view of the region of in-gap defect bands and the lower part of the CB for MTO:Mn^4+^ in the spin state *S* = 3/2 is presented in Figure 7a.

The structure of DOS for the MTO:Mn^4+^ system with spin *S* = 1/2 is more complex (Figure 6 and Figure 7b). As in the case of *S* = 3/2, the Mn atoms produce two in-gap unoccupied defect bands (in the region of 3.1 eV for α-electrons and 3.6–3.9 eV for β-electrons), which are located slightly further from the CB than in the case of spin *S* = 3/2. However, one more in-gap defect band—an occupied manganese band that forms the Fermi level—is clearly visible near the VB in Figure 6. This band consists of Mn states mixed with oxygen states. The minimum energy difference between this band (the Fermi level) and first unoccupied state (in-gap unoccupied defect band) is 3.6 eV (indirect transitions for β-electrons). At the same time, for α-electrons, the energy transition between the last occupied state (0.3 eV below the Fermi level) and the first unoccupied state (in-gap unoccupied defect band) is 3.4 eV (indirect transitions). In addition to the defect in-gap bands, formed by Mn, Figure 6 and Figure 7b reveal significant modification in the upper part of the VB and the lower part of the CB. Overall, the top of the VB (α-electron states) is formed by a mix of Mn and oxygen states, while the bottom of the CB (both α- and β-electron states) is formed by Mn, Ti and oxygen states, which leads to a decrease in the effective value of the band gap. However, the width of these modified zones will decrease with decreasing dopant concentration, and perhaps these zones may be transformed into separate in-gap defect bands that are located in close proximity to the VB and CB. Figure 7 allows us to compare in detail the unoccupied in-gap defect bands and the lower parts of the CB for the cases of *S* = 3/2 and *S* = 1/2.

### 3.3. Vibrational Properties, IR and Raman Spectra

The full vibrational analysis that we have performed in this study includes the calculation of TO phonon modes in the Γ-point, IR and Raman scattering intensities, simulation of one-phonon IR and Raman spectra, and analysis of atomic vibrations in vibrational modes.

The unit cell of perfect MTO (sg91) consists of 28 atoms, so this system has 84 normal lattice vibrations. Three of these are acoustic vibrations; the others are optical, distributed over 61 nondegenerate (A and B) and double degenerate (E) modes. There are no silent modes (neither IR- nor Raman-active) in this set; some of the modes are both IR- and Raman-active simultaneously (sg91 is non-centrosymmetric group that allows simultaneous IR and Raman activity of modes). In our case, A and B modes are either IR- or Raman-active modes, while E modes are both IR- and Raman-active. The calculated TO vibrational modes are located in the range of 129–744 cm^−1^.

Due to the large number of modes (although many have low intensity and minimal contribution to the spectrum), the density of modes with significant intensity in some spectral regions can be quite high. When calculating the spectrum, convolution of the intensities with the resolution function (in our simulations, the Lorentzian function) blurs the system of spectral peaks, since several modes located close to each other in frequency can contribute to the same spectral peak. Therefore, it is not always possible to establish a one-to-one correspondence between a peak in the spectrum and the mode appropriate to it. Figure 8 presents IR and Raman spectra of perfect MTO with different damping factors (FWHM of resolution function)—8 and 30 cm^−1^. Obviously, the second option is less informative, but it is closer to the experimental conditions.

Let us first consider the vibrations of individual groups of atoms (equivalent in symmetry) in perfect MTO and determine the modes with the maximum contribution of these vibrations. For these purposes, we have used the isotopic substitution method that allows us to change (increase) the relative atomic masses of certain atoms in a system and compare the vibrational frequencies calculated using standard atomic masses and the new isotopic masses. The magnitude of the frequency shifts of vibrational modes (isotopic shift) allows us to estimate the relative contribution of these atoms to a specific vibrational mode. In this study, we have increased the relative masses of atoms by approximately 10% (for Mg, the old mass was 24.0 and the new mass 26.4; Ti: 47.9 and 52.7; O: 16.0 and 17.6). The isotopic shift of frequencies was calculated for five different cases of changing the isotopic mass: (i) four Mg atoms in tetrahedra (Mg__tetr_); (ii) four Mg atoms in octahedra (Mg__oct_); (iii) four Ti atoms; (iv) eight O1 atoms; and (v) eight O2 atoms.

We will start with isotopic substitution of cations. The largest contributions of vibrations of Mg__tetr_ atoms are observed in the modes of the low and medium frequency ranges. The maximum absolute values of isotopic shifts are demonstrated by two Raman-active modes: the isotopic shift is Δν = 8.2 cm^−1^ for B1 mode with a frequency ν_0_ = 276 cm^−1^ and Δν = 7.3 cm^−1^ for A1 mode with ν_0_ = 388 cm^−1^. The intensity of the B1 mode in Raman scattering is close to zero, and the intensity of A1 mode is less than 10% of the maximum intensity; therefore, these two modes actually make no contribution to the Raman spectrum. At the same time, the vibrations of Mg__tetr_ atoms give significant contribution (Δν = 5.1 cm^−1^) to the IR-active mode A2 (ν_0_ = 364 cm^−1^) with the third intensity among IR-active modes, and, as a consequence, to the IR spectrum (Figure 8a). Although Mg__tetr_ atoms vibrate in the A2 mode in all three directions, the main contribution is from vibrations in the *xy*-plane.

The vibrations of Mg__oct_ atoms mainly contribute to the modes in the mid-frequency range. The IR-active mode A2 (ν_0_ = 433 cm^−1^) exhibits the maximum isotopic shift (Δν = 10.1 cm^−1^). Its intensity is about 17% of the maximum intensity of the IR-active modes. Interestingly, vibrations of Mg__oct_ atoms are realized only in the *xz*- or *yz*-planes. Both IR- and Raman-active mode with ν_0_ = 417 cm^−1^ has isotopic shift Δν = 8.8 cm^−1^. The main vibrations of Mg__oct_ atoms in this mode occur in the *xy*-plane. The intensity of this mode in IR scattering is about 58% of the maximum intensity of IR-active modes, so its contribution to the IR spectrum is significant (Figure 8a). In contrast, the intensity of this mode in Raman scattering is negligible. The Raman-active mode B2 (ν_0_ = 457 cm^−1^) has the third-highest value of isotopic shift (Δν = 8.1 cm^−1^); however, its intensity in Raman scattering is also negligible.

The largest isotopic shifts due to changes in the mass of Ti atoms are observed in three Raman-active modes: B2 (ν_0_ = 555 cm^−1^, Δν = 8.8 cm^−1^), B2 (ν_0_ = 309 cm^−1^, Δν = 6.3 cm^−1^) and A1 (ν_0_ = 303 cm^−1^, Δν = 5.7 cm^−1^). The vibrations of Ti atoms occur only in the *xz*- or *yz*-planes in B2 modes, and in A1 mode only along the *x*- or *y*-axes. Of these three modes, only mode A1 has a noticeable intensity in Raman scattering (the relative intensity of the line corresponding to this mode is 0.16, while the maximum intensity of the Raman line is taken to be 1), and the corresponding peak is presented in the Raman spectrum (Figure 8b). Note also IR-active mode A2 (ν_0_ = 617 cm^−1^, Δν = 5.5 cm^−1^). The intensity of this mode in IR scattering is about 15% of the maximum intensity of IR-active modes, and a corresponding peak is observed in the IR spectrum (Figure 8a). The vibrations of Ti atoms in this IR mode again occur only in the *xz*- or *yz*-planes.

Now we will consider vibrations of oxygen atoms. In general, the main contributions of oxygen vibrations are observed in the high-frequency range of vibrational spectra. Vibrations of eight O1 atoms give the maximum contribution to the formation of Raman-active mode A1 (ν_0_ = 744 cm^−1^, Δν = 20.7 cm^−1^). It is the last TO vibrational mode, with maximum frequency; this mode has maximum intensity in Raman scattering and is responsible for the emergence of the dominant peak in the Raman spectrum (Figure 8b). The vibrations of the O1 atoms in this mode occur mainly in the *xy*-plane. Let us note four more modes with a significant contribution of O1 atoms: both IR- and Raman-active mode E (ν_0_ = 594 cm^−1^, Δν = 18.3 cm^−1^), Raman-active mode A1 (ν_0_ = 549 cm^−1^, Δν = 17.6 cm^−1^), IR-active mode A2 (ν_0_ = 617 cm^−1^, Δν = 16.6 cm^−1^) and Raman-active mode B1 (ν_0_ = 690 cm^−1^, Δν = 16.0 cm^−1^). Mode E has the second-highest intensity among IR-active modes (92% of the maximum intensity of IR-active modes) and forms a corresponding peak in the IR spectra (Figure 8a). At the same time, the Raman intensity of this mode is almost zero. In this mode, vibrations of O1 atoms occur in all directions. Raman intensities of A1 and B1 Raman-active modes are close to zero. IR-active mode A2 has an intensity about 15% of the maximum intensity of IR-active modes and takes part in the formation of a corresponding peak in the IR spectra (together with one more vibrational mode with ν_0_ = 618 cm^−1^ and a similar intensity).

Vibrations of O2 atoms are most pronounced in the following modes: Raman-active mode B1 (ν_0_ = 556 cm^−1^, Δν = 21.3 cm^−1^), both IR- and Raman-active mode E (ν_0_ = 618 cm^−1^, Δν = 20.9 cm^−1^), two Raman-active A1 modes (ν_0_ = 501 cm^−1^, Δν = 18.7 cm^−1^ and ν_0_ = 642 cm^−1^, Δν = 17.9 cm^−1^) and IR-active mode A2 (ν_0_ = 560 cm^−1^, Δν = 15.4 cm^−1^). The Raman scattering intensity of the B1 mode is close to zero, and the vibrations of O2 atoms in this mode occur predominantly in the *xz*- or *yz*-planes. The intensity of the E mode in IR scattering is about 14% of the maximum intensity of IR-active modes, while the intensity in Raman scattering is 0.19. The E mode forms a spectral peak in the IR spectrum together with the IR-active mode A2 (ν_0_ = 617 cm^−1^), which we discussed above when considering the vibrations of O1 atoms. In Raman scattering, the E mode forms an isolated peak in the Raman spectrum (Figure 8b). O2 atoms vibrate in this mode in all directions. The intensities of Raman-active A1 modes are small: 0.04 and 0.08 for modes with ν_0_ = 501 cm^−1^ and ν_0_ = 642 cm^−1^, respectively. In IR scattering, the intensity of mode A2 is about 29% of the maximum intensity of IR-active modes, which allows this mode to form a separate peak in the IR spectrum (Figure 8a). The moving of O2 atoms in this mode occurs in the *xz*- or *yz*-planes.

We have considered here motion of atoms and contributions these atoms to vibrational modes. Now we will briefly review the main (most intense) peaks in the IR and Raman spectra of perfect MTO and summarize the conclusions about which modes and, accordingly, the vibrations of which groups of atoms make the main contribution to the formation of these peaks. To separate the peaks more clearly, we will consider the spectra obtained with FWHM = 8 cm^−1^ (Figure 8). We will start with the Raman spectrum, since it has a simpler structure. As mentioned, this spectrum has a dominant peak at 744 cm^−1^, which is formed by the Raman-active mode. The main contribution to this mode comes from vibrations of O1 oxygen atoms, and to a lesser extent by O2 atoms, while Mg atoms make the smallest contribution. Let us consider three more peaks in the Raman spectra (Figure 8b). The first is the peak at 420 cm^−1^. It is formed by the Raman-active mode with the main contribution from vibrations of O2 oxygen atoms, as well as O1 and Mg__oct_ atoms. The smallest contribution comes from vibrations of Ti atoms. The second peak, at 303 cm^−1^, is formed by the Raman-active mode with the main contribution of Mg__oct_ and Ti atoms. Mg__tetr_ atoms make the smallest contribution. One more peak (618 cm^−1^), which we mentioned earlier, is formed by the E mode, with the main contribution coming from vibration of the O2 and Ti atoms. Mg atoms make the smallest contribution.

We will highlight here the five most intense peaks in the IR spectrum (Figure 8a). The most intense peak is at 347 cm^−1^. This peak is formed by E mode, the main contribution to which is provided by oxygen atoms (first of all by O2) and Ti atoms. The next most intense peak is located nearby (364 cm^−1^) and is due to the IR-active vibrational mode. The main contributions to this mode are made by O2, Mg__tetr_ and Ti atoms. O1 atoms give the smallest contribution. The smallest of the five most intense peaks is located at 417 cm^−1^. This peak is formed by E mode, which we already mentioned when discussing the vibrations of Mg__oct_ atoms. These atoms make the largest contribution to this mode. The oxygen vibrations (O2 atoms, and to a lesser extent O1 atoms) also play a role in the formation of this mode. The next peak we will discuss is located at 436 cm^−1^. This peak corresponds to mode E. O1, Mg__oct_ and O2 atoms provide the main vibrational contribution to this mode. The smallest contribution comes from vibrations of the Ti atoms. Finally, the fifth peak in the spectrum is located at 594 cm^−1^. The origin of this peak is also associated with the E vibrational mode (interestingly, four of the five most intense peaks in the IR spectrum are associated with E modes). Vibrations of O1 atoms provide the main contribution to this mode (we have already mentioned this mode when discussing the vibrations of O1 atoms).

We have considered vibrational properties of perfect tetragonal MTO and the IR and Raman spectra of this material. Now we will briefly discuss the vibrational properties and spectra of the defective MTO:Mn^4+^ system. For comparison, the IR and Raman spectra of perfect MTO and MTO:Mn^4+^ are presented in Figure 9. The structure of these spectra is complex (except for the fact that the Raman spectra have a dominant peak). These spectra contain many closely located peaks, and it will probably be very difficult to distinguish between the spectra of the perfect system and the system with a defect. Therefore, we will not provide here the same examination of the vibrational properties of the entire system as for the perfect MTO but will limit ourselves to an analysis of the vibrations of only the Mn atom. Due to the low symmetry of the defective MTO:Mn^4+^ system (sg3), all calculated TO vibrational modes in this system are nondegenerate, and all these modes are both IR- and Raman-active. The modes are located in the range of 135–751 cm^−1^.

The largest isotopic shift due to the Mn atom (the old and new masses of Mn are 54.9 and 60.4, respectively) is predicted for the mode with ν_0_ = 556 cm^−1^ (Δν = 2.6 cm^−1^). The intensities of this mode in both IR and Raman scattering are very small. The vibrations of the Mn atom occur in the *xz*-plane. The next mode with a noticeable contribution of Mn vibrations has a frequency ν_0_ = 315 cm^−1^ and Δν = 2.2 cm^−1^. The IR intensity of this mode is small, while the Raman intensity is 0.11, and the corresponding peak is presented in the Raman spectrum (Figure 9b). The Mn atom vibrates in this mode exclusively along the *y*-axis. One more mode has the isotopic shift Δν = 2.2 cm^−1^; it is the mode with ν_0_ = 176 cm^−1^. IR and Raman intensities of this mode are also small. The vibrations of the Mn atom again occur in the *xz*-plane. Note that another 17 modes have the isotopic shift due to the Mn atom in the range of 1–2 cm^−1^. Our analysis reveals that the Mn atom as a dopant does not significantly affect the vibrational properties of the system and does not create vibrational modes that could be crucially manifested in the IR and Raman spectra.

### 3.4. Dielectric and Optical Properties

After examining the vibrational properties in the previous subsection, in this one we will turn to the analysis of some dielectric and optical properties of perfect MTO and MTO:Mn^4+^.

A crystal of the tetragonal crystal system is optically anisotropic; it has one main optical axis, which coincides with the principal symmetry axis (*z*-axis) of the crystal. In the tetragonal phase, MTO is an optically uniaxial crystal exhibiting birefringence with ordinary and extraordinary refractive indices. This means that the permittivity and refractive index tensors are completely described by two independent components: ordinary *n_o_* (*n_xx_* = *n_yy_*) and extraordinary *n_e_* (*n_zz_*).

Our calculations yield the following values for the components of the electronic (high-frequency) permittivity (also called the optical dielectric tensor or relative optical permittivity): *ε^el^_xx_* = *ε^el^_yy_* = 3.60 and *ε^el^_zz_* = 3.68. These values reflect only the electronic response and contain the contribution of purely electronic polarizability (the displacement of electron shells relative to nuclei under the influence of a light wave). The square root of these values gives the components of the refractive index: *n_xx_* = *n_yy_* = 1.90 and *n_zz_* = 1.92. The magnitude of birefringence is *Δn* = *n_e_* – *n_o_* = 0.02. Thus, our calculations reveal that the tetragonal phase of MTO is optically positive, and in this crystal the electronic polarizability along *z*-axis is slightly higher than in the *xy*-plane. Note that an experimental value of the refractive index of 2.05 is reported for bulk cubic MTO crystals [45].

Now we will discuss the ionic contributions to the dielectric response of system and consider the important dielectric parameter—the static dielectric tensor *ε*(0). The static dielectric tensor is a second-rank tensor; for MTO, the diagonal elements of the tensor are the sum of the contributions of electronic polarizability *ε_el_* and the ionic (lattice) vibrations *ε_vib_*(0), while the off-diagonal elements are equal to zero. Calculation of the vibrational contribution to the static dielectric tensor gives the following results: *ε^vib^_xx_*(0) = *ε^vib^_yy_*(0) = 8.72 and *ε^vib^_zz_*(0) = 13.07. These values represent the sum of the vibrational contributions of all IR-active modes. The maximum contribution (4.14) to *ε^vib^_zz_*(0) comes from the A2 mode (ν_0_ = 364 cm^−1^), which is formed mainly by vibrations of the O2, Mg__tetr_ and Ti atoms. In turn, this mode leads to the appearance of the second most intense peak in the IR spectrum (see Figure 8a). The doubly degenerate mode E (ν_0_ = 347 cm^−1^) gives the maximum contribution (2.63) to *ε^vib^_xx_*(0) = *ε^vib^_yy_*(0). Oxygen and Ti atoms make the main contribution to this mode, and this mode corresponds to the highest peak in the IR spectrum (Figure 8a).

Let us summarize the results on the vibrational contribution to the static dielectric tensor of perfect MTO. The difference between the components is *ε^vib^_zz_*(0) – *ε^vib^_xx_*(0) = 4.35, i.e., the crystal retains the anisotropy sign inherent at the electronic level. This is because the phonon modes of cation–oxygen bonds in MTO make a greater contribution to polarizability along *z*-axis than in the *xy*-plane. The components of total static permittivity are as follows: *ε_xx_*(0) = *ε_yy_*(0) = 12.32 and *ε_zz_*(0) = 16.75. This means that the electronic contribution is approximately 20–30% of the total static permittivity of the material. The remainder is associated with the ionic (lattice) polarization.

Now we briefly consider the dielectric properties of the MTO:Mn^4+^. Since the calculation of this system was performed with low symmetry (sg3), the dielectric tensors are no longer diagonal, and the *xx*-component is not equal to the *yy*-component. At the same time, both the difference between the *xx*- and *yy*-components and the off-diagonal elements are quite small. Doping with Mn resulted in a slight increase in the values of the electron permittivity: *ε^el^_xx_* = 3.71, *ε^el^_yy_* = 3.71, and *ε^el^_zz_* = 3.79. The vibrational contribution to the static dielectric tensor, conversely, decreased: *ε^vib^_xx_*(0) =7.64, *ε^vib^_yy_*(0) = 7.64, and *ε^vib^_zz_*(0) = 10.43. The maximum contribution to *ε^vib^_zz_*(0) comes from the mode with ν_0_ = 383 cm^−1^. This mode creates a peak of maximum intensity in the IR spectrum (see Figure 9a). The mode with ν_0_ = 359 cm^−1^ gives the maximum contribution to *ε^vib^_yy_*(0), and the mode with ν_0_ = 361 cm^−1^ to *ε^vib^_xx_*(0). Both these modes form the second most intense peak in the IR spectrum (Figure 9a). The total static permittivity of MTO:Mn^4+^ is slightly less than for perfect MTO: *ε_xx_*(0) = 11.35, *ε_yy_*(0) = 11.35, and *ε_zz_*(0) = 14.22.

### 3.5. Elastic Properties

The elastic behavior of a crystal lattice is described by its matrix of second-order elastic constants *C_ij_*. Such a symmetric elastic matrix of pure MTO, i.e., a tetragonal crystal system with the point group *D_4_*(*422*), has six independent non-zero constants, whereas the elastic matrix for MTO:Mn^4+^ (a monoclinic crystal system) contains 13 independent components [46]. All these elastic constants, as well as the bulk modulus (*B_H_*), shear modulus (*G_H_*), Young’s modulus (*E_H_*) and Poisson’s ratio (*ν_H_*) in the Hill averaging scheme [47], for both systems are collected in Table 4 (for comparison, Table 4 also demonstrates the calculated data for the perfect tetragonal MTO from Ref. [19]). Note that the Hill moduli are obtained as the mean of the respective Voigt (as an upper limit) and the Reuss (as a lower limit) moduli.

The necessary and sufficient Born elastic stability conditions for the crystals of the tetragonal crystal system with the point group *D_4_*(*422*) are as follows [46]:

*C*_11_ > |*C*_12_|, 2*C*_13_*C*_13_ < *C*_33_(*C*_11_ + *C*_12_), *C*_44_ > 0, *C*_66_ > 0, (2)

and MTO satisfies these four conditions. Moreover, the ratio *B_H_*/*G_H_* ≈ 2.16 satisfies Pugh’s criterion (Pugh’s ratio *B*/*G* > 1.75) and demonstrates that tetragonal MTO behaves in a ductile manner [48]. The value of Poisson’s ratio *ν_H_* = 0.299 confirms this conclusion (Frantsevich criterion of behavior in a ductile manner *ν_H_* > 0.26).

Analyzing Table 4, we can conclude that a 25% Mn concentration has virtually no effect on the elastic properties of the crystal. All elastic constants change slightly, and these changes occur in different directions. Those components of the elasticity tensor of MTO:Mn that were equal to 0 in perfect MTO (*C*_15_, *C*_25_, *C*_35_, and *C*_46_) differ little from 0, and those that became independent components in MTO:Mn (*C*_22_, *C*_23_, *C*_55_) differ little from the corresponding components in MTO.

In previous studies [10,11], we calculated the Debye temperature of K_2_SiF_6_ and Cs_2_SiF_6_ crystals based on the obtained bulk modulus and shear modulus of these materials. Using the same algorithm [10,11,49], the data from Table 4, the density (3561 kg/m^3^) and the molar mass (0.16 kg/mol) of perfect MTO, we obtained for MTO a Debye temperature of 699 K, which is very close to the value of 697.2 K obtained in Ref. [19].

## 4. Conclusions

Detailed first-principles calculations of the structural, electronic, vibrational, dielectric and elastic properties of the Mg_2_TiO_4_ tetragonal phase, pure and Mn^4+^-doped, were performed in the present paper. All structural characteristics, such as lattice constants and interionic distances, were thoroughly calculated and compared with the corresponding experimental data, yielding excellent agreement between the theoretical and experimental data. Peculiar features of the chemical bonding between cations and oxygen ions were analyzed by comparing electronegativities and calculated effective charges of the ions involved in bond formation. The normal vibrations frequencies were calculated, including the effect of isotopic cation and oxygen substitution on the frequencies of the normal modes. Since the considered host can be used as a red-emitting phosphor after doping with the Mn^4+^ ions, the influence of such doping on all above-mentioned properties was modeled by replacing one Ti^4+^ ion in a unit cell by one Mn^4+^ ion and performing the corresponding calculations. Special attention was paid to the changes in the Mn–O distances if compared to the Ti–O distances and to the location of the impurity ion energy levels in the host’s band gap. The obtained data can serve as a valuable reference for the comprehensive analysis of similar spinel compounds, both pure and doped with the Mn^4+^ ions, and will be used in calculations of the luminescent properties of Mn dopant in upcoming studies.

## Figures and Tables

**Figure 1:**
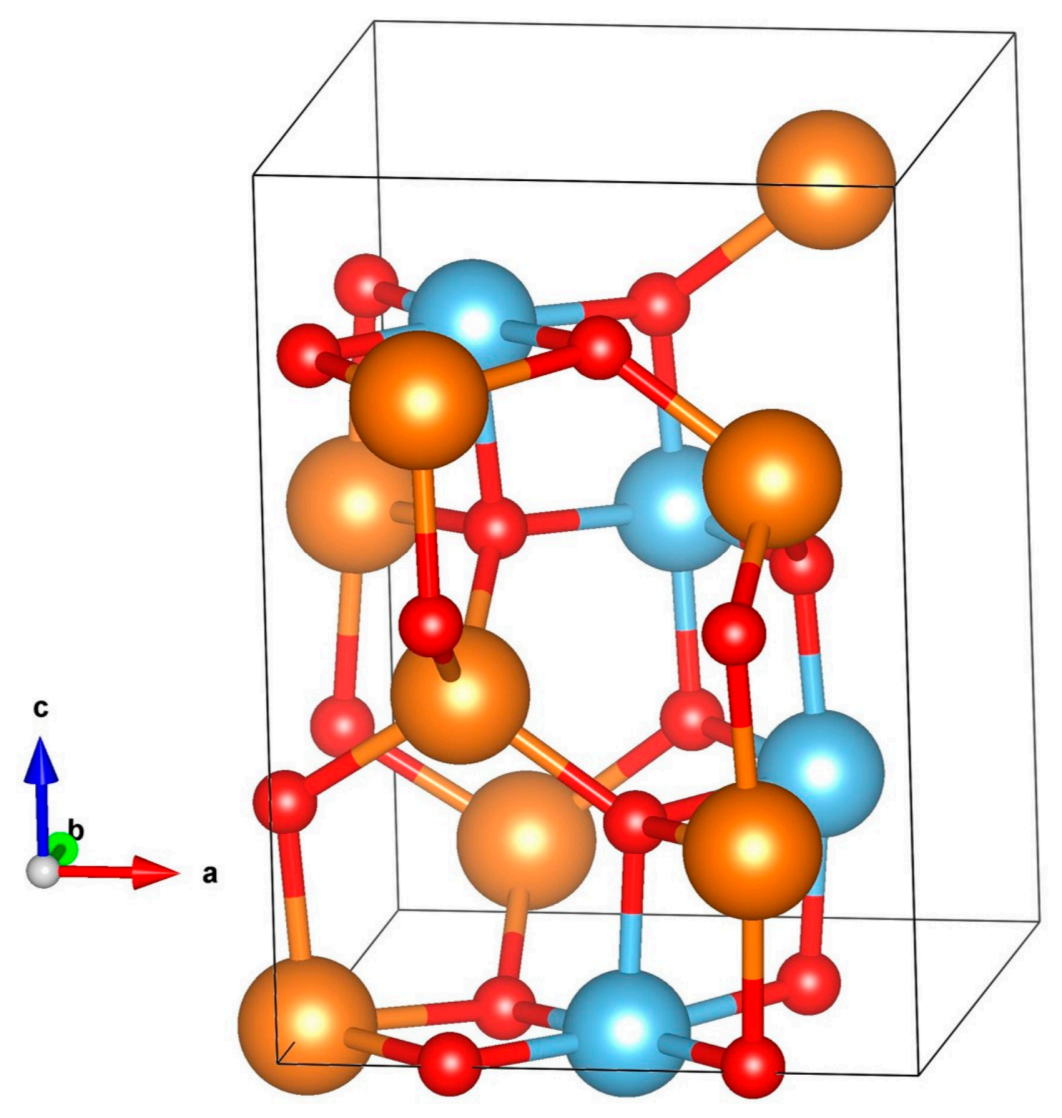
Simulated unit cell of perfect MTO in the tetragonal phase (28 atoms). Mg ions are orange balls; Ti—blue balls; O—red balls. The black lines represent the unit cell.

**Figure 2:**
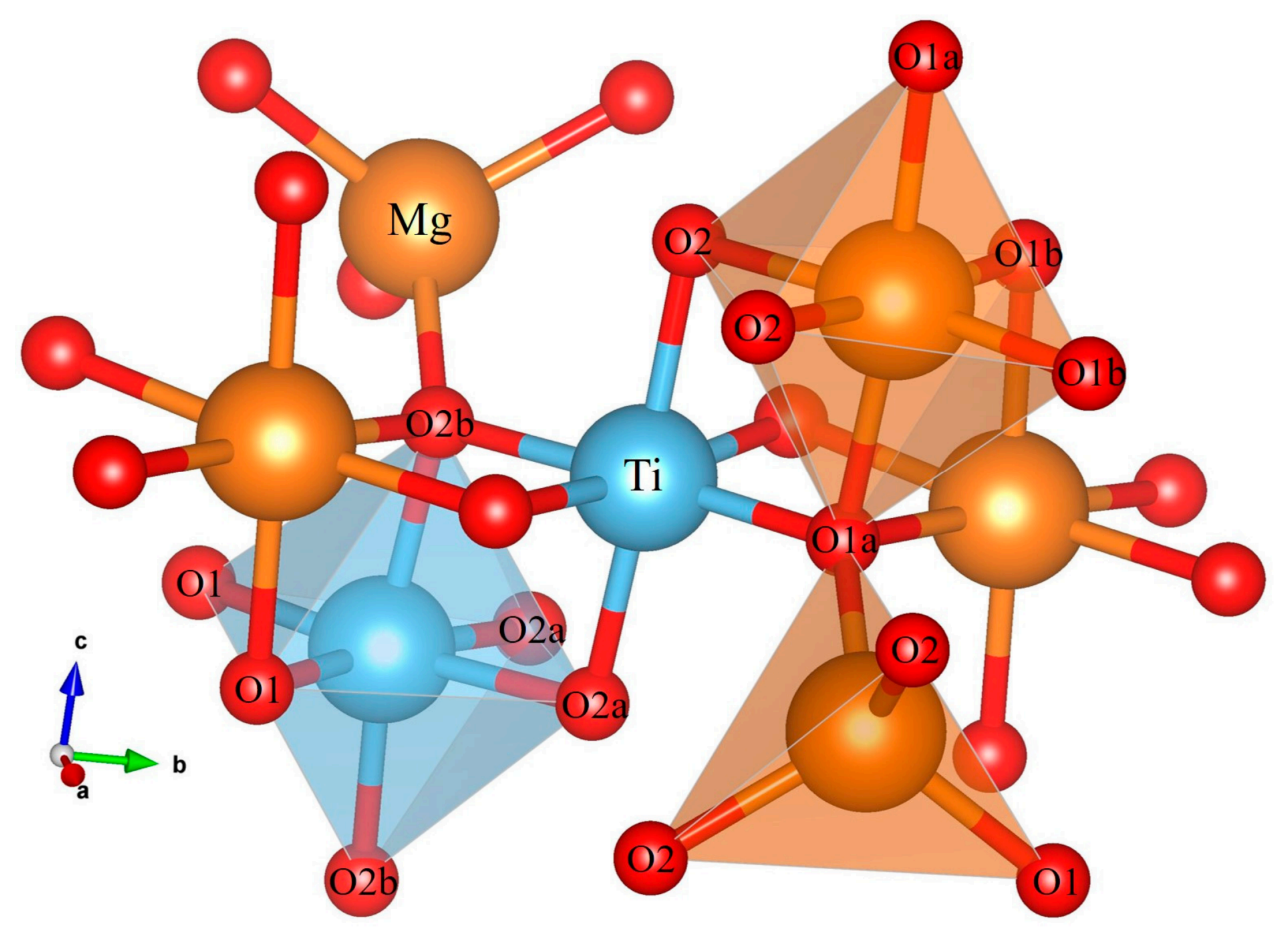
A part of the crystal structure of MTO with Mg and Ti cations in the centers of octahedra and Mg cations in the centers of tetrahedra. One tetrahedron around the Mg cation and two octahedra around the Mg and Ti cations are highlighted.

**Figure 3:**
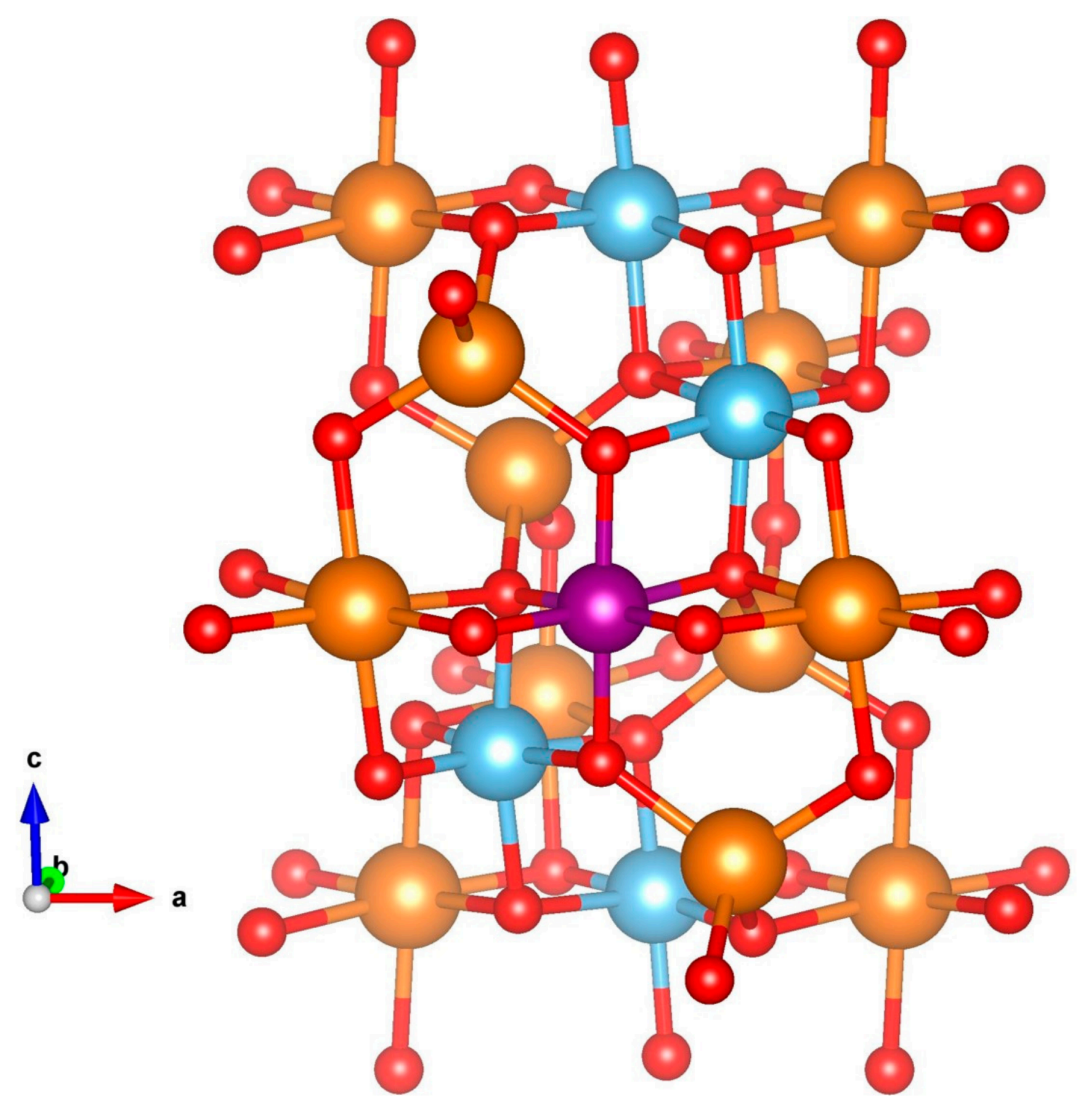
A part of the crystal structure of MTO:Mn^4+^, where one of the four Ti atoms in the unit cell is replaced by a Mn atom. Mn atom—violet ball.

**Figure 4:**
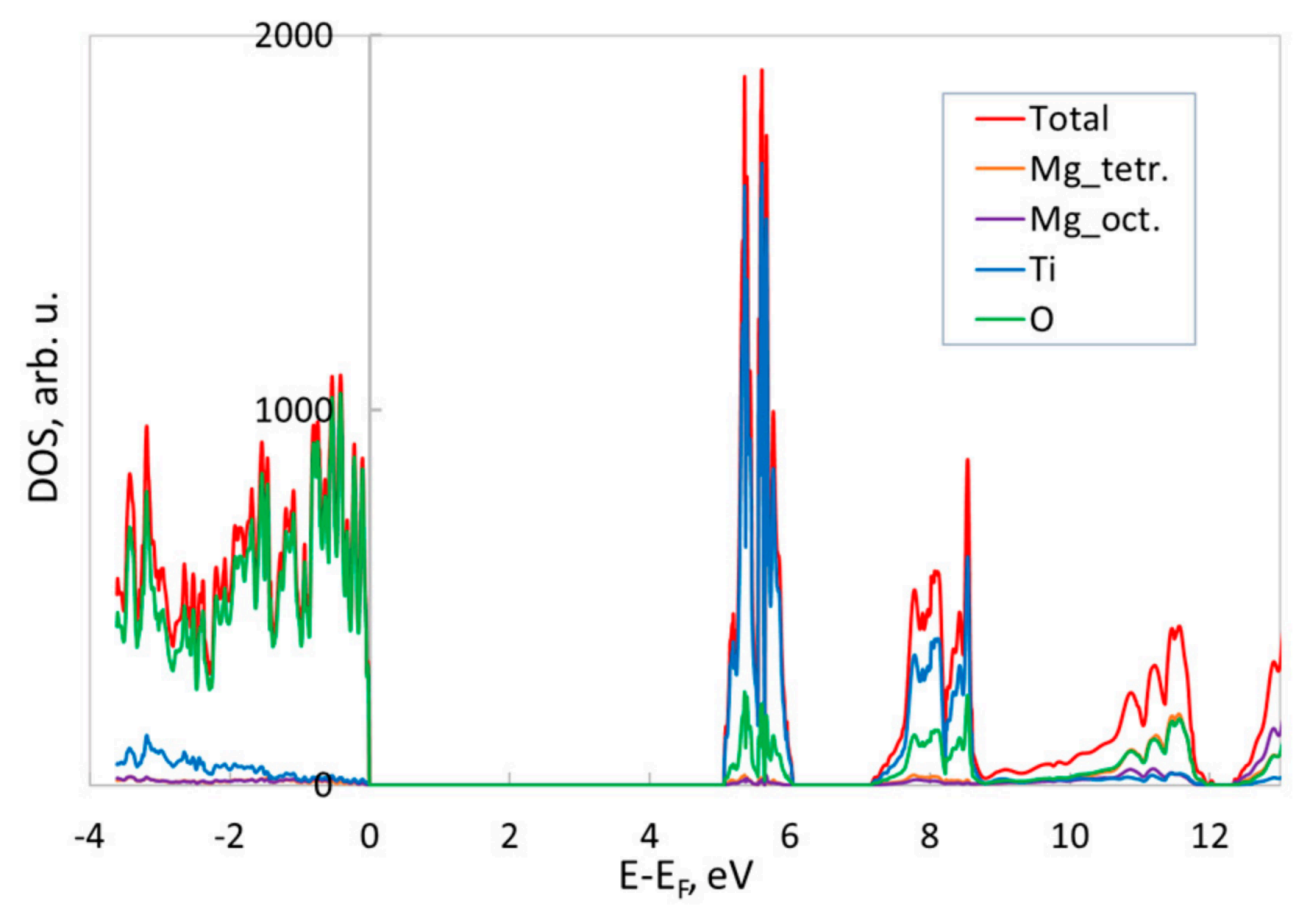
Projected and total electronic DOSs of perfect MTO sg91. The contributions of Mg ions in tetrahedral and octahedral positions are presented separately. The zero energy value corresponds to the Fermi level.

**Figure 5:**
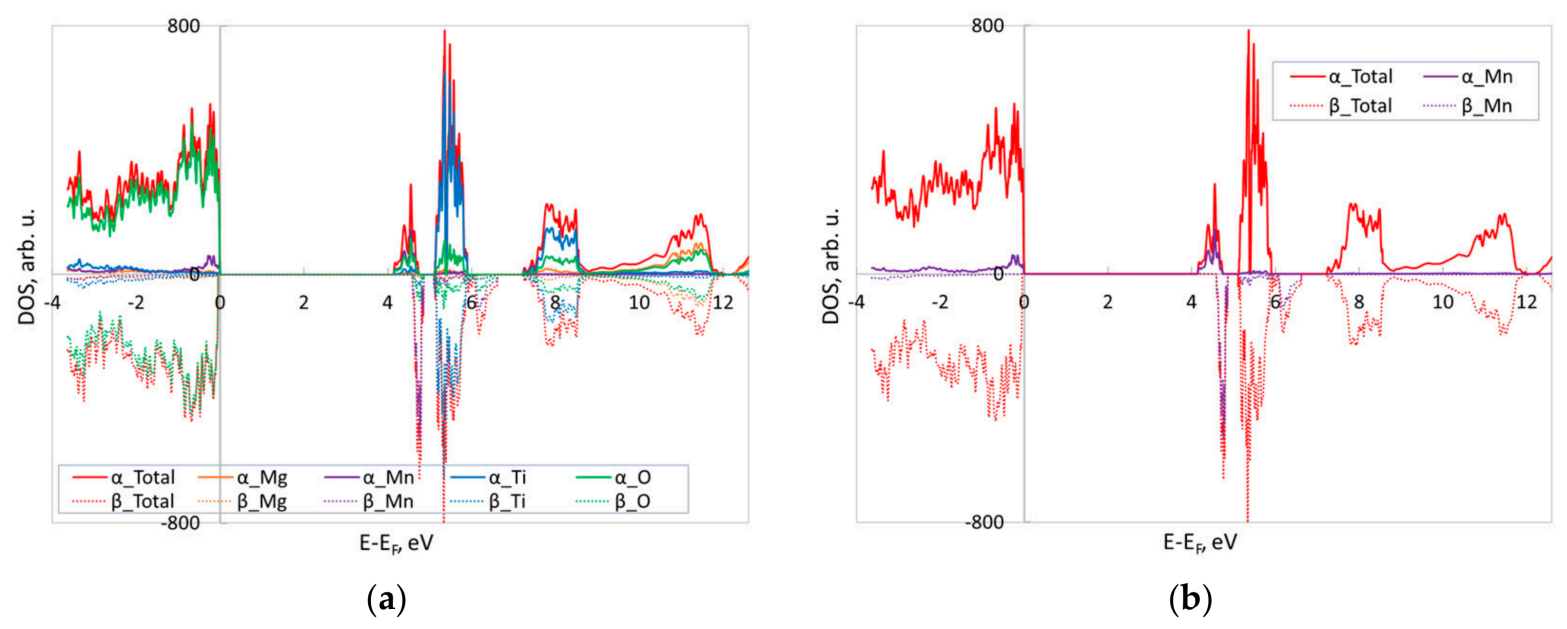
DOS of MTO:Mn^4+^ *S* = 3/2, sg3. The zero energy value corresponds to the Fermi level. (**a**) The total DOS and the projected DOS of the Mg, Ti, O and dopant Mn atoms. (**b**) The total DOS and the projected DOS for the Mn atom only.

**Figure 6:**
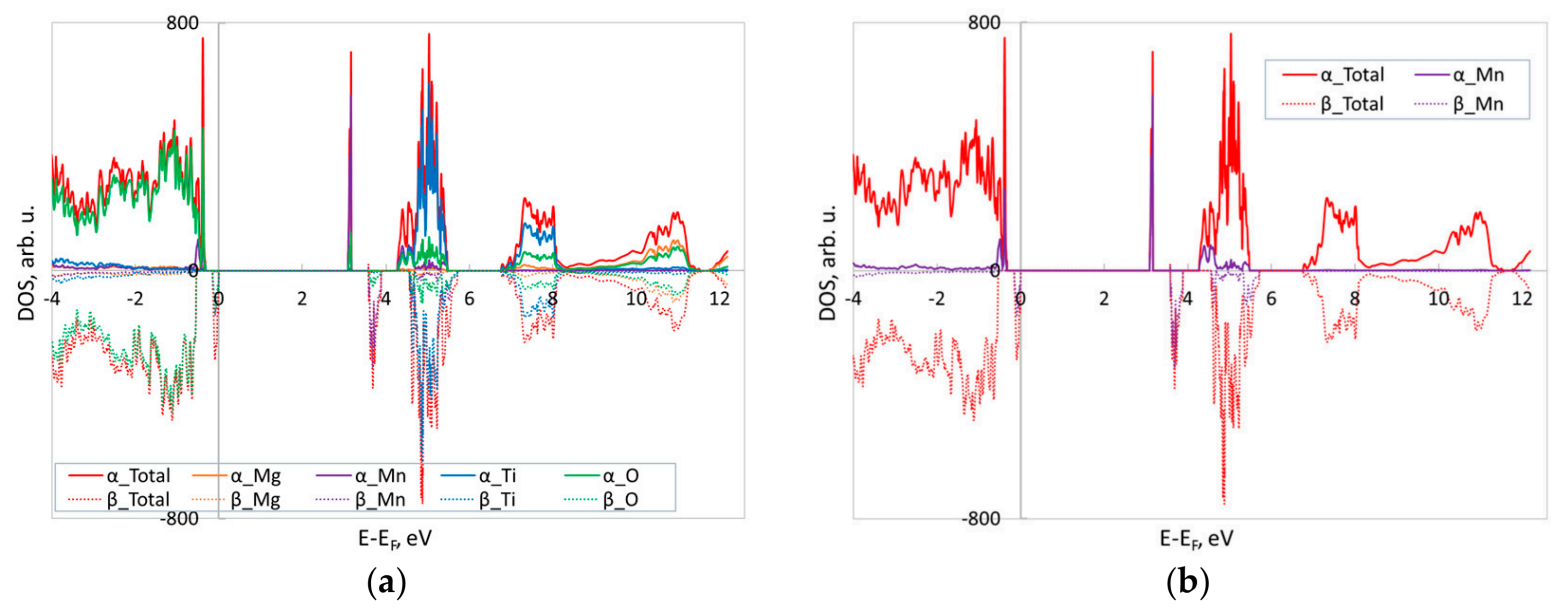
DOS of MTO:Mn^4+^ *S* = 1/2, sg3. The zero energy value corresponds to the Fermi level. (**a**) The total DOS and the projected DOS of the Mg, Ti, O and dopant Mn atoms. (**b**) The total DOS and the projected DOS for the Mn atom only.

**Figure 7:**
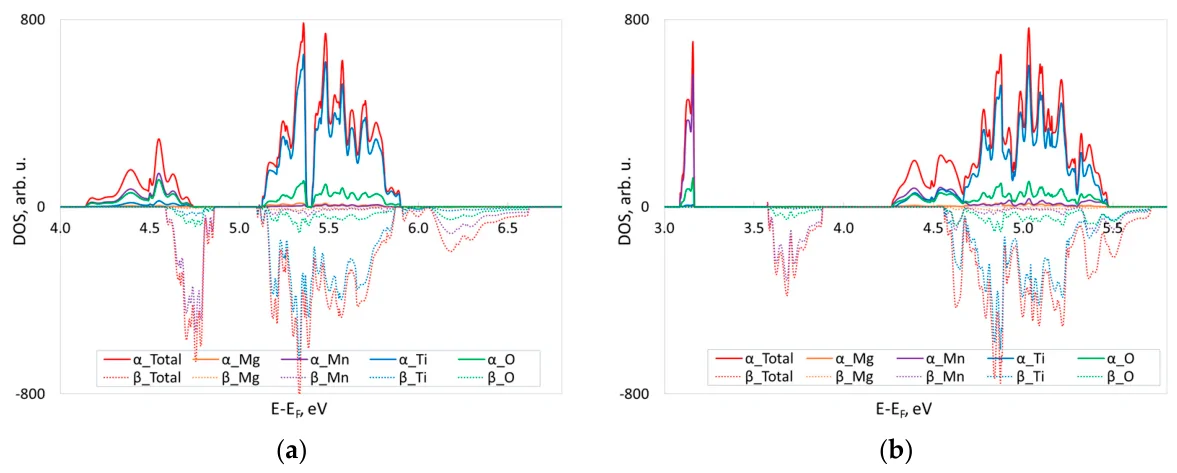
DOS of MTO:Mn^4+^, sg3. The zero energy value corresponds to the Fermi level. (**a**) *S* = 3/2. (**b**) *S* = 1/2.

**Figure 8:**
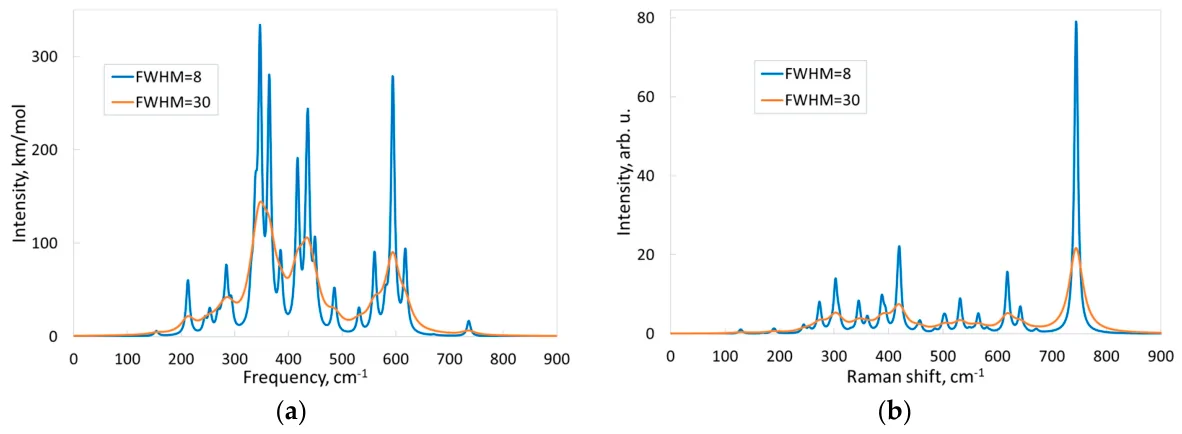
Calculated one-phonon spectra of perfect MTO with two different resolution functions (FWHM is equal to 8 and 30 cm^−1^). (**a**) Infrared spectra. (**b**) Raman spectra.

**Figure 9:**
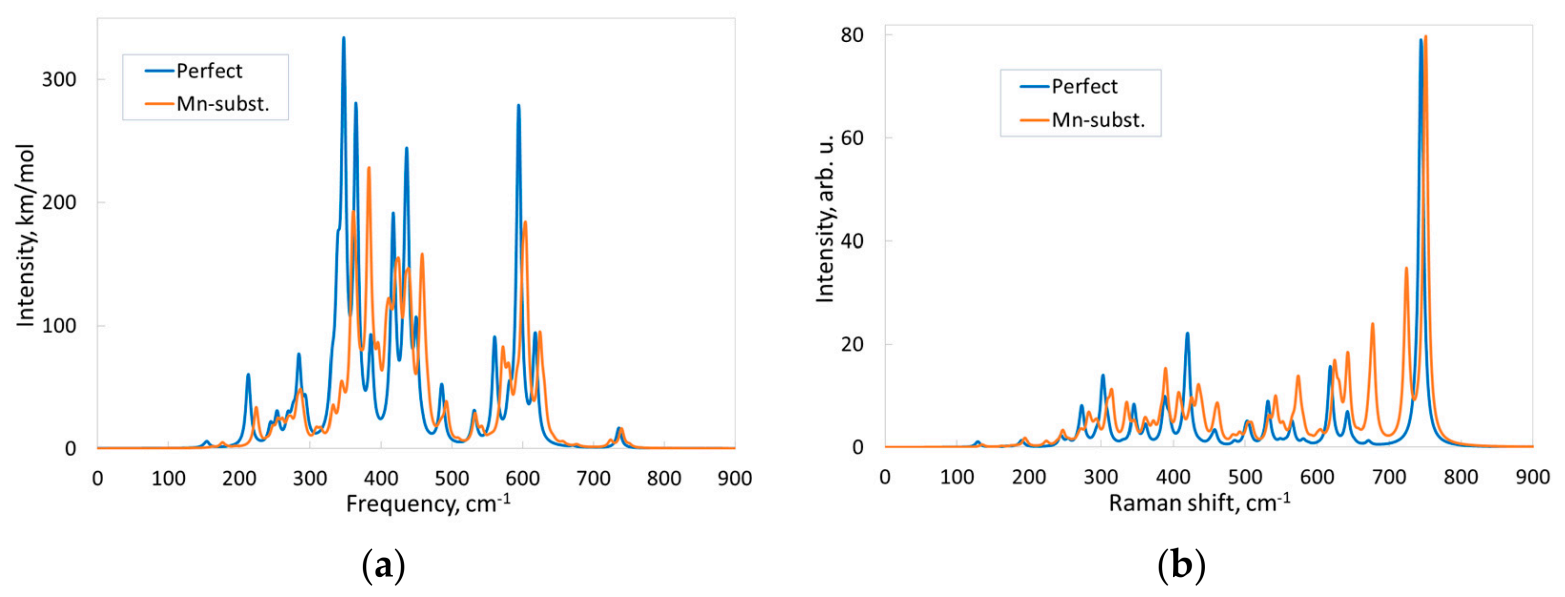
Calculated one-phonon spectra of perfect MTO and MTO:Mn^4+^ (*S* = 3/2, sg3). (**a**) Infrared spectra. (**b**) Raman spectra.

**Table 1:** Calculated variable parts (Calc.) of atomic coordinates in comparison with experimental values (Expt.) for perfect tetragonal MTO.

Atoms and Their Wyckoff Positions	Coordinates	Calc (This Work)	Expt [14]
Mg__tetr_, 4c			
	*x*	0.2518	0.2518
Mg__oct_, 4a			
	*y*	0.2494	0.2502
Ti, 4b			
	*y*	0.2381	0.2391
O1, 8d_1_			
	*x*	−0.0282	−0.0264
	*y*	0.7365	0.7368
	*z*	0.2519	0.2526
O2, 8d_2_			
	*x*	0.5176	0.5188
	*y*	0.2620	0.2610
	*z*	0.2329	0.2329

**Table 2:** Selected calculated structural parameters (Calc.) of the perfect MTO along with corresponding experimental data (Expt.). The numbers in parentheses are the discrepancies between calculated and measured values (as a percentage).

	Calc.	Expt. [14] *	Expt. [13] *
*a* = *b*, Å	5.968	5.977 (−0.15)	5.975 (−0.12)
*c*, Å	8.371	8.416 (−0.53)	8.414 (−0.51)
*d*[Mg–O1]_tetr_, Å	1.981	1.981 (0.00)	1.981 (0.00)
*d*[Mg–O2]_tetr_, Å	1.984	1.995 (−0.55)	1.986 (−0.10)
*d*[Mg–O1a]_oct_, Å	2.086	2.090 (−0.19)	2.093 (−0.33)
*d*[Mg–O1b]_oct_, Å	2.053	2.065 (−0.58)	2.039 (0.69)
*d*[Mg–O2]_oct_, Å	2.097	2.088 (0.43)	2.111 (−0.66)
*d*[Ti–O1], Å	1.887	1.903 (−0.84)	1.912 (−1.31)
*d*[Ti–O2a], Å	2.041	2.039 (0.10)	2.038 (0.15)
*d*[Ti–O2b], Å	1.957	1.968 (−0.56)	1.967 (−0.51)
<*d*[Mg–O]_tetr_>, Å	1.983	1.988 (−0.25)	
<*d*[Mg–O]_oct_>, Å	2.079	2.081 (−0.10)	
<*d*[Ti–O]_oct_>, Å	1.962	1.970 (−0.41)	
*V_tetr_*(Mg), Å^3^	4.00	4.03 (−0.74)	
*V_oct_*(Mg), Å^3^	11.72	11.78 (−0.51)	
*V_oct_*(Ti), Å^3^	9.95	10.07 (−1.19)	
<*d*[O1–(3Mg + 1Ti)]>	2.002	2.010 (−0.40)	
<*d*[O2–(2Mg + 2Ti)]>	2.020	2.023 (−0.15)	
*V_tetr_*(O1), Å^3^	3.83	3.88 (−1.29)	
*V_tetr_*(O2), Å^3^	3.93	3.95 (−0.51)	

* Relative deviations of the calculated values from the experimental ones (in %) are given in parentheses.

**Table 3:** A comparison of selected structural parameters of the perfect MTO and two defective systems with different spin states.

	Perfect MTO sg91	MTO:Mn^4+^ *S* = 3/2 sg3	MTO:Mn^4+^ *S* = 1/2 sg3
*a*, Å	5.968	5.948	5.948
*b*, Å	=*a*	5.949	5.948
*c*, Å	8.371	8.335	8.327
beta, °	90	90.11	90.17
*V*, Å^3^	298.14	294.92	294.57
*d*[Ti{Mn}–O1], Å	1.887 (Ti)	1.874 (Mn)	1.864 (Mn)
*d*[Ti{Mn}–O2a], Å	2.041 (Ti)	1.948 (Mn)	1.951 (Mn)
*d*[Ti{Mn}–O2b], Å	1.957 (Ti)	1.905 (Mn)	1.899 (Mn)
<*d*[Ti{Mn}–O]_oct._>, Å	1.962 (Ti)	1.909 (Mn)	1.905 (Mn)
*V_oct._*(Ti{Mn}), Å^3^	9.95 (Ti)	9.21 (Mn)	9.15 (Mn)
(O1–Ti{Mn}–O2a), °	174.5 (Ti)	176.5 (Mn)	176.4 (Mn)
(O2b–Ti{Mn}–O2b), °	171.6 (Ti)	175.6 (Mn)	175.1 (Mn)

**Table 4:** Calculated constants of elastic tensor (*C*), bulk modulus (*B*), shear modulus (*G*), Young’s modulus (*E*) and Poisson’s ratio (*ν*) for perfect MTO and MTO:Mn^4+^.

	Perfect MTO sg91	MTO:Mn^4+^ *S* = 3/2 sg3	Perfect MTO [19]
*C*_11_, GPa	287.16	286.44	268.12
*C*_12_, GPa	76.55	78.38	64.03
*C*_13_, GPa	133.72	135.80	106.11
*C*_15_, GPa	0	−0.40	0
*C*_22_, GPa	=*C*_11_	290.51	=*C*_11_
*C*_23_, GPa	=*C*_13_	133.53	=*C*_13_
*C*_25_, GPa	0	0.26	0
*C*_33_, GPa	218.61	217.40	208.19
*C*_35_, GPa	0	−1.77	0
*C*_44_, GPa	107.66	107.15	100.38
*C*_46_, GPa	0	−0.69	0
*C*_55_, GPa	=*C*_44_	106.78	=*C*_44_
*C*_66_, GPa	47.82	49.00	45.11
*B_H_*, GPa	164.49	165.43	143.99
*G_H_*, GPa	76.22	76.15	75.68
*E_H_*, GPa	198.06	198.06	193.11
*ν_H_*	0.299	0.300	

## Data Availability

The original contributions presented in this study are included in the article. Further inquiries can be directed to the corresponding authors.

## Abbreviations

The following abbreviations are used in this manuscript:

MTO	Mg_2_TiO_4_
LCAO	Linear combination of atomic orbitals
DFT	Density functional theory
sg	Space group
HF	Hartree–Fock
DOS	Sensity of states
VB	Valence band
CB	Conduction band
IR	Infrared
FWHM	Full width at half maximum
